# Umbilical Transposition in Functional Panniculectomy of the Massive Weight Loss Patient: Is It Aesthetic or Medically Necessary?

**Published:** 2012-05-21

**Authors:** Raffi Gurunluoglu, Susan A. Williams, Aslin Gurunluoglu, Jeffrey L. Johnson

**Affiliations:** ^a^Plastic and Reconstructive Surgery, Denver Health Medical Center, University of Colorado Health Sciences; ^b^Psychology and Management of Education; ^c^Surgery, Denver Health Medical Center, University of Colorado Health Sciences, Denver

## Abstract

**Background:** We review the procedures used in panniculectomy and explore the necessity of umbilical transposition when adequately treating the medical and functional problems associated with panniculus in the massive weight loss patient. **Methods:** Thirty-five consecutive patients with symptomatic panniculus after massive weight loss undergoing panniculectomy during the time period from November 2008 to October 2010 at Denver Health Medical Center were retrospectively analyzed. Inclusion criteria consisted of insurance approval for the panniculectomy. All patients had persistent skin problems in the lower abdomen. Seven patients had additional skin problems in the skin around navel and/or mid-abdomen. Eleven patients complained of difficulty in performing activities of daily living. Nine patients had a concomitant ventral hernia repair. **Results:** An infraumbilical panniculectomy was adequate in treating the medical and functional symptoms of the abdominal region in 3 patients with no need for umbilical transposition. The remaining 32 patients required a different procedure instead of only an infraumbilical panniculectomy. Among these 32 patients, 3 patients underwent panniculectomy with sacrifice of the umbilicus. Umbilical transposition following abdominal undermining was needed in the remaining 29 patients undergoing panniculectomy. **Conclusions:** Functional umbilical transposition was required to avoid unnatural displacement of the navel while treating chronic skin problems in the lower abdomen, or additional persistent skin problems around the navel or in the mid-abdomen, and to access the supraumbilical region, particularly for large ventral hernia repair during panniculectomy. Therefore, umbilical transposition in these cases was not aesthetic in nature but an integral part of achieving a functional surgical treatment.

The number of patients with symptomatic redundant skin and subcutaneous tissue has substantially increased as a result of increased weight loss procedures over the last decade.^1^ Signs and symptoms vary greatly depending on the patients' body type and amount of weight loss. However, the abdomen is typically the area of greatest concern and dysfunctionality.

In addition to aesthetic dissatisfaction, most patients who have undergone massive weight loss have a proclivity to experience medical and functional problems such as chronic intertrigo and difficulty in performing activities of daily living (ADL). Chronic skin problems are typically encountered in the lower abdomen: particularly the skin of the panniculus, the groin, or the suprapubic region. On the contrary, persistent skin problems around the navel and/or under the secondary rolls in the mid-abdomen can also be seen in some of these patients. Furthermore, most massive weight loss patients encounter functionality problems such as difficulty wearing clothes, quandaries in maintaining personal hygiene, and struggles in performing ADL. All the aforementioned issues may interfere with the patients' lifestyles to a varying degree.

The American Society of Plastic Surgeons provides functional diagnosis (*International Classification of Diseases, Ninth Revision* [*ICD-9*]) codes and procedure (*Current Procedural Terminology* [*CPT*]) codes for functional panniculectomy and redundant skin surgery. For instance, a panniculectomy to eliminate a large hanging abdominal panniculus and its associated symptoms would be considered reconstructive. On the basis of American Society of Plastic Surgeons criteria, a panniculectomy would also be considered functional (*CPT* code 15830) and reconstructive if lumbago “back pain” (*ICD-9* code 724.2) and/or panniculitis (*ICD-9* code 729.39) or intertrigo (*ICD-9* code 695.89) are associated with the excess panniculus. Moreover, a panniculectomy is considered a reconstructive procedure when performed to correct or relieve structural defects of the abdominal wall due to functional incompetence of the anterior abdominal wall. In addition, according to American Society of Plastic Surgeons recommendations, a panniculectomy is regarded as reconstructive and functional if it will improve ambulation and ADL of an individual.^2^

The functional panniculectomy involves an infraumbilical procedure where the excess skin and fat forming the hanging panniculus are removed without undermining the remaining abdominal flap. The procedure assumes that there is no need for umbilical transposition. Although this type of procedure provides an adequate treatment option for some massive weight loss patients, this particular procedure would not produce the most favorable functional outcome in others.

Oftentimes, there would be a need to remove the entire portion of excess skin and fat tissue inferior to the level of the umbilicus in order to be able to treat the chronic skin problems associated with panniculus and improve ADL. In such circumstances, if an umbilical transposition were not performed, this procedure would result in either the sacrifice of the navel or lead to the potential displacement of the navel inferiorly into an unnatural location in the lower abdomen.

Similarly, an infraumbilical panniculus resection would be inadequate for the treatment of chronic intertrigo in the skin around the navel or under the secondary rolls, which is common in massive weight loss patients. In this clinical scenario, there would be a need for a high-level panniculectomy requiring either the sacrifice of the umbilicus or its transposition.

Recently, we published a classification system in the massive weight loss patient on the basis of skin lesions and interference with ADL.^3^ We demonstrated that there are more functional procedures that may be required to treat chronic skin problems in various locations of the abdominotorso region and to improve ADL. Moreover, when treating a ventral hernia concomitantly with symptomatic panniculus, the undermining of the abdominal flap frequently used for gaining proper access would necessitate performing an umbilical transposition in most massive weight loss patients.

We retrospectively analyzed 35 consecutive patients with symptomatic panniculus after massive weight loss who underwent panniculectomy for medical and functional reasons. The goal of this article was to review the functional procedures and the necessity of umbilical transposition in these procedures when treating the medical and functional problems associated with panniculus in massive weight loss patients.

## MATERIALS AND METHODS

Thirty-five consecutive patients with symptomatic panniculus after massive weight loss who underwent panniculectomy at Denver Health Medical Center during the period from November 2008 to October 2010 were retrospectively analyzed. Inclusion criteria included insurance approval for the panniculectomy.

Patients' age ranged from 33 to 63 years. Thirty-three of our patients were women. Methods of weight loss in our patients were as follows: open gastric bypass (*n* = 30 patients, 85.7%), lifestyle and dietary changes (*n* = 4 patients, 11.4%), and lap band procedure (*n* = 1 patient, 2.69%). The average body mass index (BMI) score of the patients before weight loss measures was 50.2, with a range from 85.3 to 35.2. The average BMI score after weight loss and immediately before panniculectomy was 29.8, with a range from 46 to 19. Overall, the average weight loss for our patients was 131 lb, with weight loss ranging from 315 to 65 lb.

Seven patients (20%) had additional problems in the skin around navel and/or mid-abdomen. Eleven patients (31%) complained of difficulty in performing ADL based on findings in SF-36 (36-Item Short Form Health Survey).^3^ Nine patients (25.7%) had a ventral hernia. Thirty-two patients (91.4%) desired to preserve their navels (Table 1). In each case, a surgical treatment plan was formulated for the functional and medical problems associated with the symptomatic panniculus along with best possible aesthetic outcome.

## CASE REPORTS

### Patient 3

**Figure 1:**
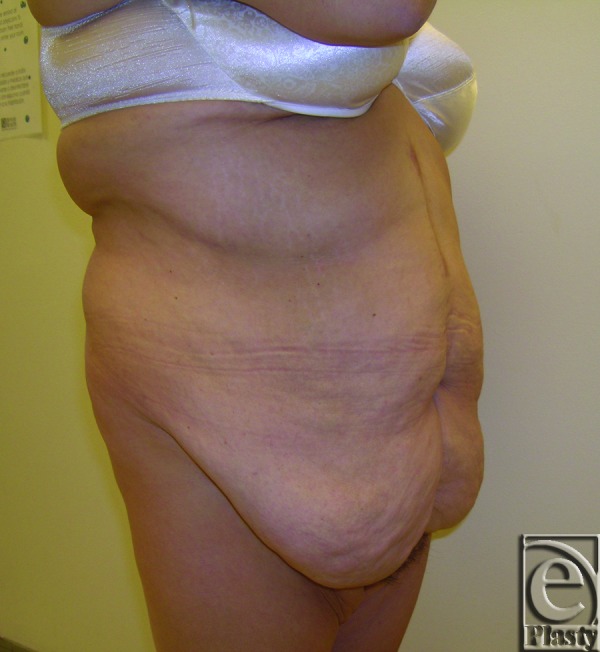
Right side view of patient 3 with a large symptomatic panniculus. A higher functional panniculectomy was required to adequately treat her skin problems in the lower abdomen and to help improve her activities of daily living. This necessitated a functional umbilical transposition.

**Figure 2:**
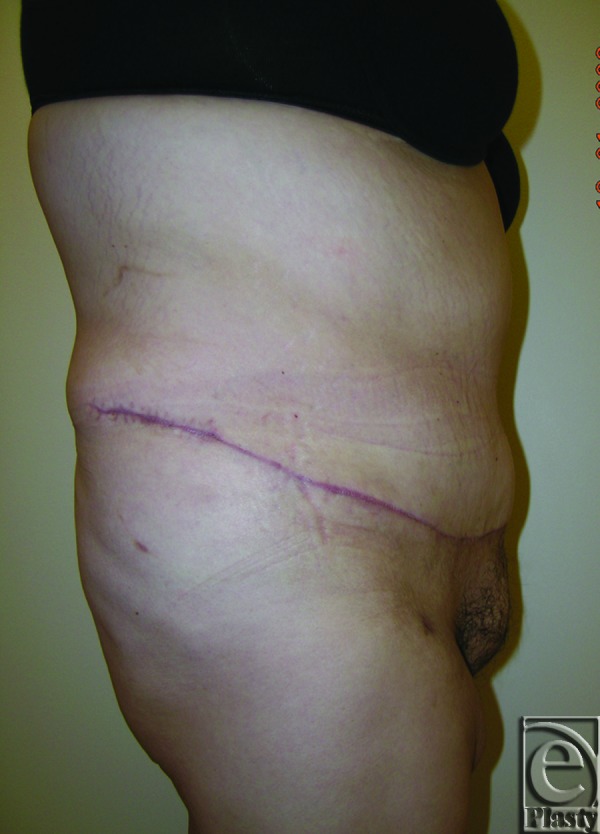
Postoperative right side view of patient 3 at 6 months.

A 37-year-old female patient presented with a large symptomatic panniculus after open gastric bypass surgery and massive weight loss (110 lb) (Fig 1). Her initial BMI was 52.67; after her weight loss, her BMI was 32.55. Chronic skin problems were confined to the lower abdomen under the large panniculus. The patient also complained of difficulty in ambulation and interference with performing ADL associated with the large panniculus. She had a strong desire to preserve her navel. She underwent a functional horizontal panniculectomy with umbilical transposition, and a total of 10.10 lb of skin and subcutaneous fat was removed. Postoperative follow-up at 6 months showed no evidence of skin problems and improved ADL, based on her SF-36 score (Fig 2).

### Patient 8

**Figure 3:**
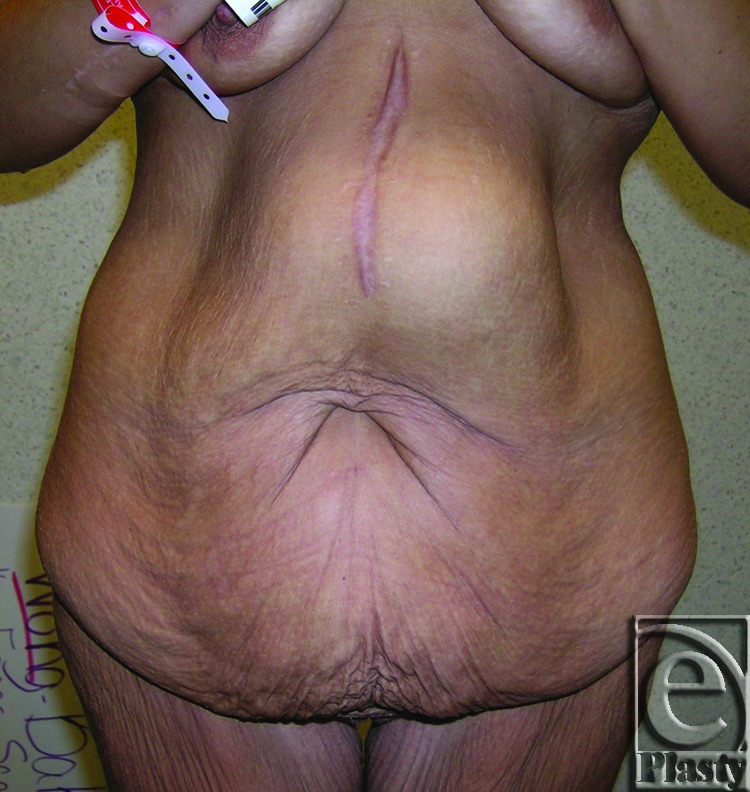
Preoperative frontal view of patient 8 with a symptomatic panniculus. Her skin problems were confined to the skin around the navel as well as in the lower abdomen under the panniculus. She also had an incisional ventral hernia.

**Figure 4:**
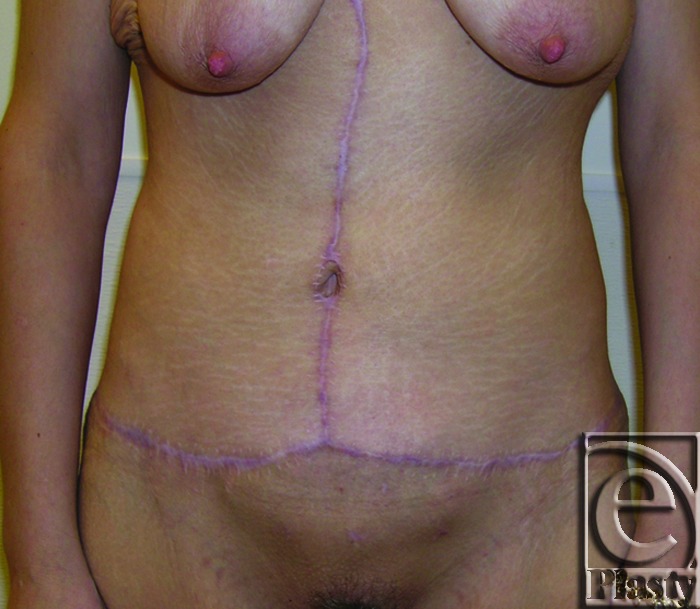
Postoperative frontal view of patient 8 at 12 months.

A 34-year-old female patient complained of frequent infection and rashes in the skin around the navel and under the pannus along the suprapubic region and both groins after open gastric bypass surgery and massive weight loss (122 lb). Her BMI decreased from 44.38 to 24.60. Of note, she had a large incisional ventral hernia associated with her gastric bypass surgery (Fig 3). She wanted to preserve her navel. She underwent a functional horizontal panniculectomy with umbilical transposition. Concomitant hernia repair was performed. A minimal vertical skin and subcutaneous excision overlying the supraumbilical hernia was also performed because of compromised perfusion. A total of 3.6 lb of skin and subcutaneous fat was removed. Twelve-month follow-up showed resolution of her skin problems and no evidence of hernia recurrence (Fig 4).

### Patient 10

**Figure 5:**
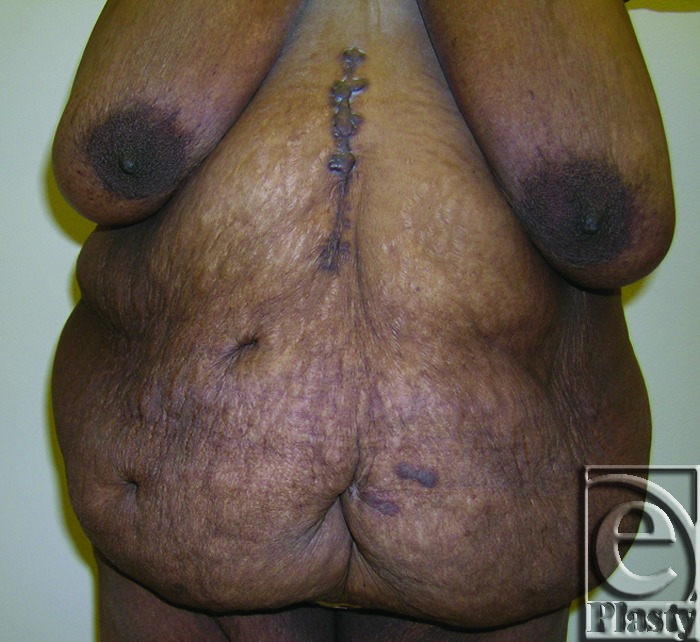
Preoperative frontal view of patient 10 showing a large symptomatic panniculus.

**Figure 6:**
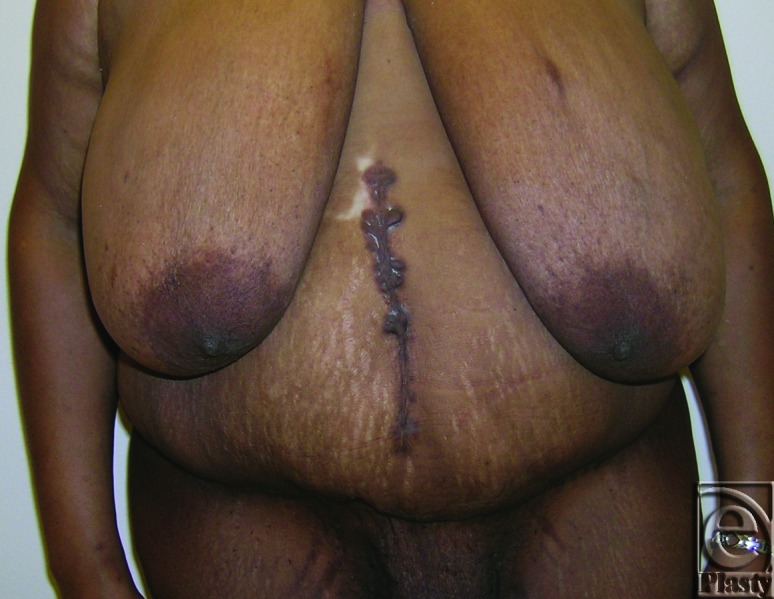
Postoperative frontal view of patient 10 at 9 months after a functional panniculectomy and umbilical sacrifice.

A 45-year-old female patient presented with a large panniculus and associated chronic skin problems in the lower abdomen after massive weight loss (140 lb) following open gastric bypass surgery. This resulted in a decrease of her BMI from 58.87 to 34.84. She complained of a significant difficulty in ambulating and carrying out her ADL associated with the large panniculus (Fig 5). This was supported by her preoperative SF-36 score. She was indifferent to preservation of her navel. She underwent a horizontal panniculectomy with the sacrifice of her navel. A total of 11.0 lb of skin and subcutaneous fat was removed. There was no evidence of skin problems at 9 months postoperatively. Patient also reported improved ADL, as observed in her postoperative SF-36 score (Fig 6).

### Patient 16

**Figure 7:**
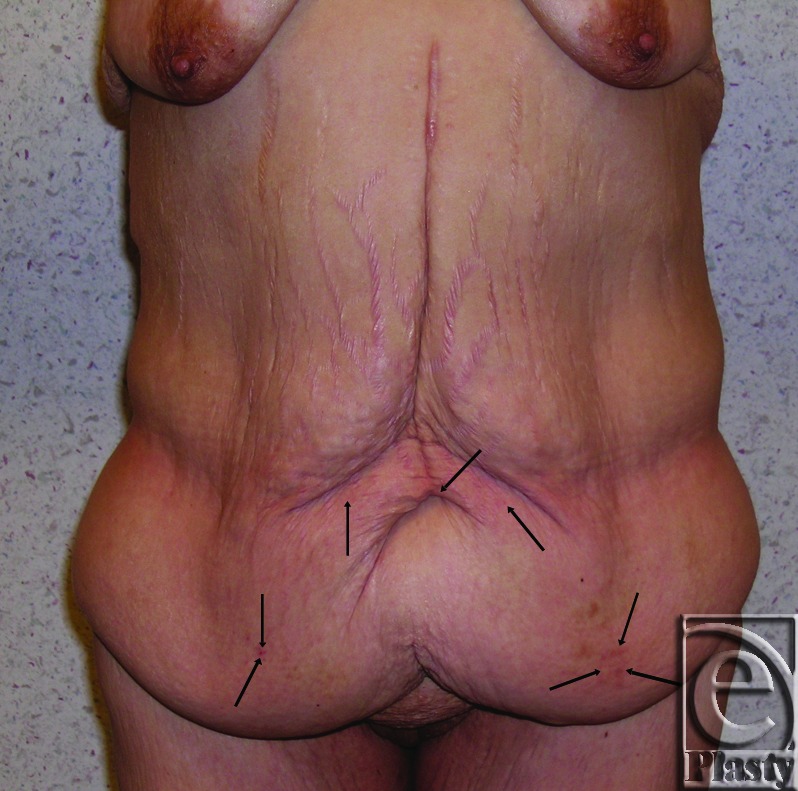
Preoperative frontal view of patient 16 whose chronic skin problems were confined to the skin around the navel (arrows) and in the lower abdomen (arrows).

**Figure 8:**
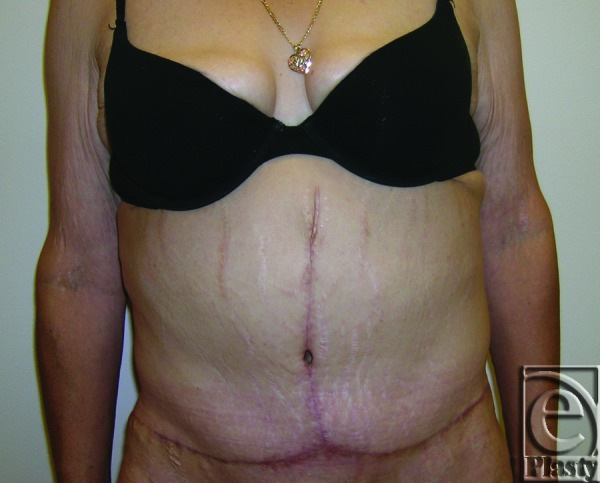
Postoperative frontal view of patient 16 at 9 months after a functional panniculectomy, a minimal vertical redundant skin excision, and umbilical transposition.

A 37-year-old female patient presented with skin problems confined to her lower abdomen and around the navel after open gastric bypass surgery and massive weight loss (130 lb). There was also skin irritation in the vertical redundant folds around the navel (Fig 7). Her BMI decreased from 46.67 to 23.99. She complained of interference with her ADL. She desired to preserve her navel and thus underwent a functional horizontal panniculectomy, minimal excision of vertical excess skin and subcutaneous fat from the supraumbilical region, and umbilical transposition. A total of 3.91 lb was removed during these procedures. Postoperative follow-up showed resolution of skin problems as early as 3 months after the surgery (Fig 8).

## RESULTS

An infraumbilical panniculectomy was adequate in treating the medical and functional symptoms of the abdominal region in 3 patients (8.6%) with no need for umbilical transposition. The remaining 32 patients (91.4%) required a different procedure instead of only an infraumbilical panniculectomy. Among these 32 patients, 3 patients underwent panniculectomy with sacrifice of the umbilicus. Umbilical transposition and abdominal undermining were needed in the remaining 29 (82.9%) patients undergoing panniculectomy. Seven patients required minimal vertical skin and subcutaneous fat excision. Nine patients (25.5%) had a concomitant incisional ventral hernia repair (Table 1).

Mean follow-up was 12.3 months after the surgery. Skin signs were resolved in all but one patient. No evidence of hernia recurrence was noted. Overall complication rate was 25.7%; there were 4 cases of hematoma development of which 2 required surgical evacuation, 2 cases of cellulitis/soft-tissue infection detected during follow-up required antibiotic treatment, 2 cases of mild wound dehiscence that resolved without surgical intervention, and 1 case of keloid scar formation. In the case of cellulitis/soft tissue infection, a small seroma with no evidence of discrete abscess was found on ultrasonography; however, it did not warrant intervention and resolved spontaneously.

## DISCUSSION

There is an enormous variability of body proportions after massive weight loss. Nevertheless, most patients desire improvement of the abdominotorso. Obviously, the procedures must be tailored to each patient's unique distribution of excess tissue and his or her individual needs. Many authors have proposed techniques to improve the aesthetic result in panniculectomy after massive weight loss.^4^^-^9 For instance, authors such as Song et al^10^ and Wallach^11^ classified the contour deformities and proposed an algorithmic approach in treating the abdominotorso region in massive weight loss patients. Abdominoplasty/panniculectomy, fleur-de-lis approach, or circumferential dermolipectomy is recommended on the basis of the quality of the skin and subcutaneous tissue and the location of the lax tissue. An umbilical transposition was performed in most of these procedures to obtain the best aesthetic outcome.

The assumption that there would be no need for umbilical transposition in massive weight loss patients when surgically treating the medical and functional signs and symptoms has been a vague subject in the literature. On the basis of our experience, we believe that if umbilical transposition were not performed, it would result in suboptimal functional treatment in most massive weight loss patients.

Our review of massive weight loss patients showed that an umbilical transposition was required in 83% of cases when surgically treating the medical and functional signs and symptoms. Hence, the authors coined the term “functional umbilical transposition.” In some patients (patients 7, 13, and 15, see Table 1), a standard infraumbilical panniculectomy resolved the lower abdominal skin problems without the need for an umbilical transposition. This surgery did not lead to the displacement of the navel to an unnatural location in the lower abdomen, nor did it require the sacrifice of the navel.

On the contrary, in some massive weight loss patients, performing just an infraumbilical skin and subcutaneous fat excision would not be sufficient to treat skin problems in the lower abdomen and/or difficulty in ADL associated with a large panniculus. Therefore, a functional transverse panniculectomy at or above the umbilicus level would be required. In our series, 22 patients with this type of clinical presentation who also desired to preserve their navels underwent umbilical transposition, as opposed to only 2 similar patients (patients 20 and 25) who were indifferent to preservation of their navels and underwent umbilical sacrifice.

Furthermore, in some massive weight loss patients, a standard infraumbilical panniculectomy would not help in treating the additional skin rashes around the navel and/or mid-abdomen, which were observed in 7 patients (patients 5, 8, 11, 16, 22, 30, and 32) of our series. Similarly, a higher level transverse panniculectomy with umbilical transposition was required in this type of patients. We were encouraged to sacrifice the umbilicus only in 1 patient (patient 22), who did not desire to preserve her navel.

In some cases of our series, minimal wedge excision of vertical redundancy was needed to supplement the treatment of the intertrigo in the vertical folds of the mid-abdomen and around the navel, as in patient 16. In the rare instance, a vertical excision was also required to remove the compromised thin skin overlying a large incisional ventral hernia after dissection to expose the hernia, as in patient 8.

Despite the increasing popularity of a laparoscopic approach, many morbidly obese patients are still offered open gastric bypass surgery. The method of weight loss for our patients was predominately open gastric bypass, influenced by the status of our hospital as a safety net hospital and our bariatric surgeons' experience. Ventral hernia is a common complication of open gastric bypass surgery and remains a serious problem. In our series, 25.7% of patients required concomitant hernia repair in addition to presenting with signs and symptoms associated with panniculus. This repair of a true hernia is covered by current insurance approval unlike diastasis recti repair commonly performed during an abdominoplasty.^12^ Literature suggests that simultaneous ventral hernia repair and panniculectomy can safely be accomplished.^13^ In fact, concurrent panniculectomy has been shown to minimize the risk of hernia recurrence.^14^ These findings have also been supported in our study.

Preexisting hernia necessitated preoperative planning for best exposure in conjunction with panniculectomy. Abdominal undermining above the level of the umbilicus was needed for access to repair the supraumbilical ventral hernia in 9 massive weight loss patients, and an umbilical transposition was considered necessary in 8 of these patients.

Overall, wound problems seem to occur frequently in massive weight loss patients, with seroma formation being the most frequent complication in most series.^15^^-^17 Several factors such as medical comorbidities, tobacco use, BMI, pannus size, type of redundant skin surgery, and the American Society of Anesthesiologists Physical Status Classification may impact the incidence of complications in this patient population.

In general, we favor the use of sharp knife for most part of the surgery and minimize the use of cautery for dissection. Nevertheless, undermining of the abdominal flap and insetting of the umbilicus in massive weight loss patients may be risky. Given the pendulous nature of the pannus in massive weight loss patients, the umbilical stalk can be elongated. In rare cases, this elongation can increase the risk of vascular compromise. Maximum care must be taken during the dissection and insetting of the umbilical stalk to avoid kinking, rotation, and undue tension. In addition, the size of abdominal aperture should be adequate to prevent incarceration of the umbilical stalk.

On the basis of our experience in massive weight loss patients, because of the redundant and stretchable properties of the abdominal skin, a minimal abdominal undermining was all that was required to allow safe umbilical transposition. This limited undermining was helpful for the reduction of the potential dead space for seroma formation. In all cases, particularly in those in whom further dissection was required for ventral hernia repair, we have taken the topmost care in obliterating the dead space with our primary quilting sutures. In addition to sharp dissection, implementation of these precautionary methods has reduced the incidence of seroma and associated wound complications as observed in this series.

The results of this review may have significant implications for patients and physicians seeking insurance coverage. Third-party payers use guidelines for medical necessity determination in the treatment of the abdominal region of massive weight loss patients. Authorization is granted on the basis of these specific guidelines. These guidelines/criteria were extensively reviewed in previous publications.^18^^,^^19^

In many instances, authorization would be restricted to a standard infraumbilical panniculectomy, assuming that an umbilical transposition is not needed in the massive weight loss patient. Conversely, the results of this study showed that an umbilical transposition was necessary in most of our cases as part of the primary functional procedure to obtain optimal functional and medical treatment. Furthermore, while many currently believe that a panniculectomy is performed purely for a functional and medical outcome, is it right for plastic surgeons to disregard a patient's desire to retain the umbilicus? We propose that there should be consideration given to the individual patient's needs and, when warranted, a *functional umbilical transposition* may be performed.

## CONCLUSIONS

Functional umbilical transposition may be required to avoid unnatural displacement of the navel while treating chronic skin problems in the lower abdomen, or additional persistent skin problems around the navel or in the mid-abdomen, and to access the supraumbilical region, particularly for large ventral hernia repair during panniculectomy. Therefore, umbilical transposition in these cases would not be aesthetic in nature but should be considered an integral part of achieving a functional surgical treatment. Because of the redundant feature of the abdominal skin, functional umbilical transposition can be achieved following minimal abdominal undermining in most cases.

## Figures and Tables

**Table 1:** Thirty-five consecutive massive weight loss patients who underwent functional panniculectomy for the treatment of medical and functional problems associated with symptomatic panniculus during November 2008 to October 2010*

Patient no.	Intertrigo around navel and/or mid-abdomen	Intertrigo lower abdomen	Interference with activities of daily living	Desire to preserve the navel	Infraumbilical functional transverse panniculectomy	Functional transverse panniculectomy	Addition of vertical component	Functional umbilical transposition	Hernia repair
1		+		+		+		+	
2		+		+		+		+	
3†		+	+	+		+		+	
4		+		+		+		+	
5	+	+		+		+		+	
6		+		+		+		+	
7		+		+	+			No need for transposition	
8†	+	+	+	+		+	+	+	+
9		+		+		+		+	+
10†		+	+	Indifferent		+		Sacrifice	
11	+	+		+		+		+	
12		+	+	+		+		+	+
13		+		+	+			No need for transposition	
14		+		+		+		+	+
15		+		+	+			No need for transposition	
16†	+	+	+	+		+	+	+	
17		+		+		+		+	+
18		+		+		+		+	+
19		+		+		+		+	
20		+		+		+		+	
21		+		+		+		+	
22	+	+	+	Indifferent		+	+	Sacrifice	+
23		+		+		+		+	
24		+		+		+		+	
25		+	+	Indifferent		+		Sacrifice	
26		+	+	+		+		+	
27		+		+		+	+	+	
28		+		+		+	+	+	
29		+	+	+		+	+	+	+
30	+	+		+		+		+	
31		+		+		+	+	+	
32	+	+	+	+		+		+	
33		+	+	+		+		+	
34		+		+		+		+	+
35		+		+		+		+	
Total	7 (20)	35 (100)	11 (31)	32 (91.4)	3 (8.6)	32 (91.4)	7 (20)	29 (83)	9 (25.7)

*The values given are number (percentage) of umbilical transposition.

†Case reports presented in the article.
